# Ipsilateral Foot Drop After Leg Traction on Fracture Table for Mid-Shaft Femur Fracture Nailing: A Rare Case Report

**DOI:** 10.7759/cureus.43826

**Published:** 2023-08-20

**Authors:** Jehad A Alzahrani, Ahmed A Alabdali, Mohammed O Albariqi

**Affiliations:** 1 Orthopaedic Surgery, King Fahad General Hospital, Albaha, SAU; 2 Orthopaedic Surgery, Prince Mansour Military Hospital, Altaif, SAU; 3 Orthopaedic Surgery, King Fahad General Hospital, Jeddah, SAU

**Keywords:** iotrogenic injury, traction table complication, drop foot, femur shaft fracture, sciatic nerve injury

## Abstract

Femoral shaft fracture, one of the most common orthopaedic injuries, is usually treated with intramedullary nailing. During the operative procedure patients are placed on a traction table. Traction tables facilitate the procedure but are associated with some risk. Here we are sharing a case of a 35-year-old male healthy young patient who sustained a foot drop post nailing of femoral shaft fracture on a traction table. This patient has had some recovery in six weeks but is still not fully recovered. We think traction tables are a very helpful tool but carry some risks that should be kept on mind for every surgeon, and for the patients too.

## Introduction

Sciatic nerve injuries have been reported post intramedullary nailing (IMN) of a femur fracture via several different causes, including iatrogenic clamp malposition [1], the nerve being trapped at the fracture site [2], or secondary to post-operation hematoma formation [3]. Sciatic nerve injury can also be noticed in a delayed setting post-IMN secondary to anticoagulants [4] or as a complication of hemi lithotomy postion on orthopedic traction tables. Herein, we report an ipsilateral foot drop in a healthy young patient following the IMN of a femur midshaft transverse fracture using a traction table. To the best of our knowledge, no similar case has been reported with this finding; however, a similar traction table-related foot sensory neurapraxia has previously been reported [5].

## Case presentation

**Figure 1:**
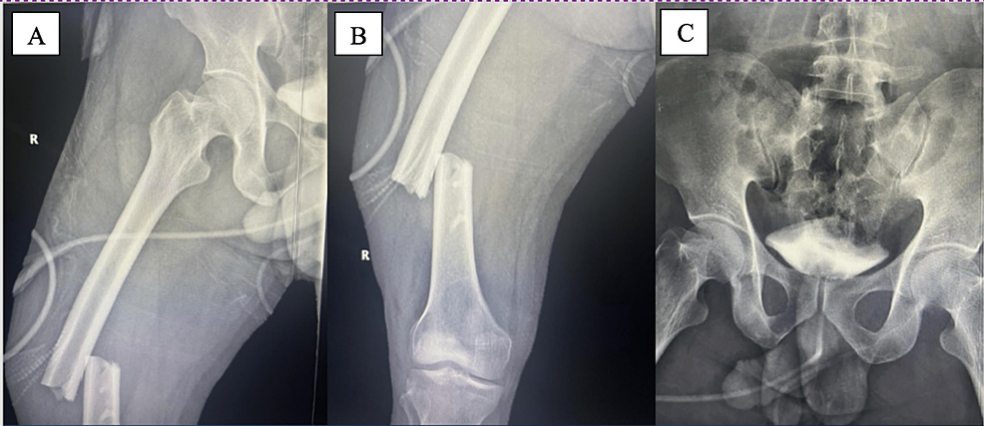
Preoperative radiographs of the right femur, revealing a closed femoral shaft fracture. Anteroposterior view (A and B); anteroposterior view of the pelvis (C).

A 35-year-old male with a body mass index (BMI) of 28 who has no past medical history sustained a road traffic accident (RTA) following a frontal collision with a concrete structure while driving his small sedan car at a speed of around 100 km/h. He was wearing a seat belt, was not ejected from the car, and had no loss of consciousness or head trauma. A primary survey was inconclusive, while his secondary survey revealed a right femur midshaft fracture (Figure 1). A distal neurovascular assessment also showed no abnormality. In the emergency department, skeletal traction of the proximal tibia was applied, and a re-examination revealed that the patient had a normal and fully intact distal neurovascular status.

**Figure 2:**
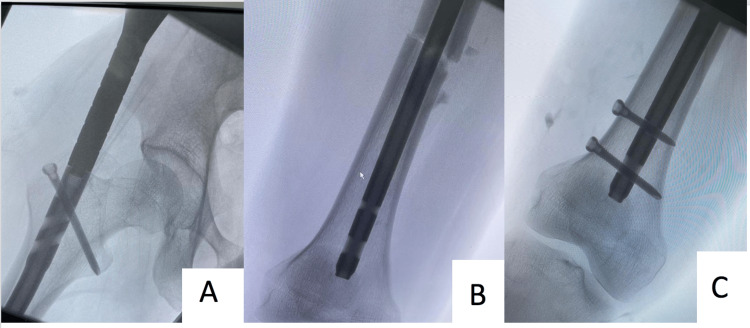
Intraoperative radiographs illustrate the anteroposterior view of the right femur. Trochanteric entry nail with proximal interlocking screw (A); fracture site (B); two distal interlocking screws (C).

**Figure 3:**
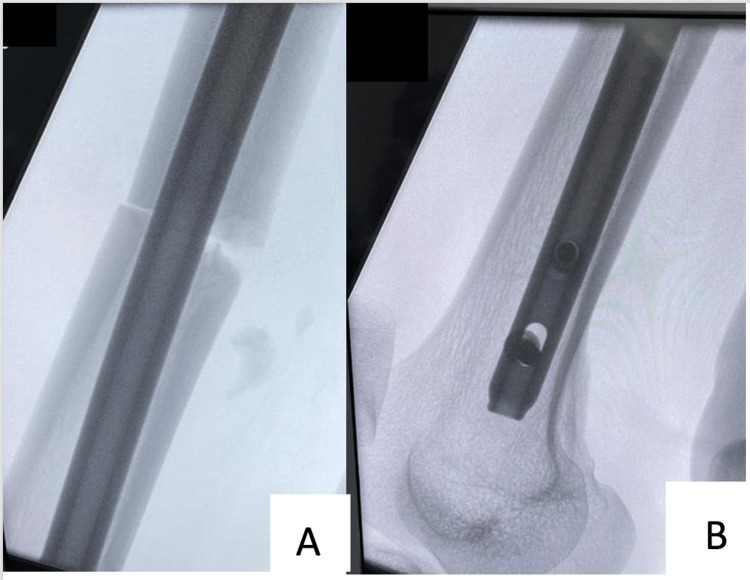
Intraoperative radiographs illustrate the lateral view of the right femur fracture site (A) and two distal interlocking screws (B).

**Figure 4:**
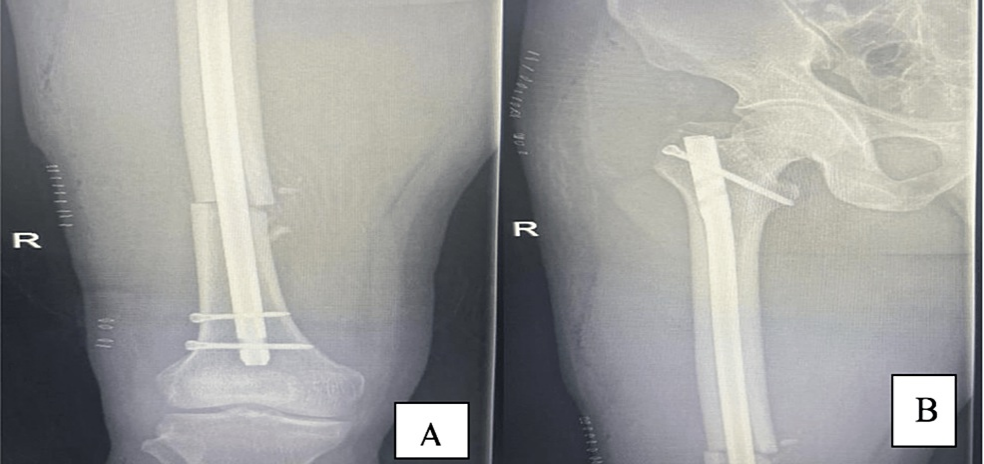
Postoperative radiographs illustrate the anteroposterior view of the right femur (A and B).

On the fourth day of admission, the patient was operated on, following his pre-operation examination, which showed normal intact distal neurovascular status. The patient was placed on a traction table using a supine position with bilateral boots and traction, where we position the fractured leg with traction in a 20° hip flex position. The unaffected leg is positioned in a 30° hip extension position on the other side of the post in a traction boot, and closed reduction and fixation of the fracture were performed by IMN using a trochanteric entry through a lateral approach (Figures 2, 3) without intraoperative difficulties (Figure 4). The day one postoperative examination revealed that the patient had an ipsilateral foot drop, yet his sensation remained intact; thus, an ankle foot orthosis (AFO) was applied. The patient was discharged home on day four postoperation. After two weeks, the patient was seen in the clinic and did not demonstrate any improvement. The patient was also observed in the clinic one month later and had regained some ankle dorsiflexion but had still not fully recovered.

## Discussion

Traction tables have been used for a long time in orthopedics as reduction devices for femur fractures because they can provide advantages, such as a higher traction force and stability, and easier fluoroscopy [6]. However, there are also some drawbacks to their use, such as fracture malreduction, nerve injuries, and soft tissue injuries. Moreover, new studies have demonstrated better results and no difference in the outcome between IMN with and without a traction table, respectively [7]. 

Traction table-related complications in the sciatic nerve are commonly linked to the position rather than the traction table; thus, it is widely reported to be a normal leg injury, which is only secondary to the hemi lithotomy position. This seems to be an accepted idea among orthoepedic surgeons, while it is also recommended to avoid the lithotomy position and minimize the risks of sciatic nerve and pudendal nerve injuries [8].

Our case involves an ipsilateral foot drop, which was noticed when performing nailing on a traction table. We believe that it was this traction that caused an injury, which, to our knowledge, has never been previously mentioned. Furthermore, the injury might be related to longer operation/traction times.

In an effort to reduce further traction-related complications, it is recommended to release the traction immediately after applying the interlocking screw. Additionally, some authors recommend applying the tourniquet safety protocol to the traction, which includes periodic release every 120 minutes since applying 70 mmHg of pressure for this amount of time can result in microscopic damage. Moreover, a perineal post with a diameter greater than 10 cm may also be used [9].

## Conclusions

This rare case report can potentially raise awareness among orthopedic surgeons regarding traction table-related complications. Using traction tables has several benefits, yet it also possesses some risks, which surgeons should be aware of, including fracture malrotation, pudendal nerve neurapraxia, and perineal soft tissue injury with under padded perineal post, or as in our case, a foot drop resulting from prolonged traction, which has only gradually started resolving after six weeks.
